# Effect of self-acupressure on middle ear barotrauma associated with hyperbaric oxygen therapy: A nonrandomized clinical trial

**DOI:** 10.1097/MD.0000000000025674

**Published:** 2021-04-30

**Authors:** Jen-Ming Chen, Zheng-Nan Lu, Re-Wen Wu, Kuo-Wei Bi, Chun-Ting Liu

**Affiliations:** a Department of Chinese Medicine, Kaohsiung; b Department of Orthopedic Surgery, Kaohsiung Chang Gung Memorial Hospital and Chang Gung University College of Medicine, Kaohsiung; c Graduate Institute of Integrated Medicine, College of Chinese Medicine, Research Center for Chinese Medicine & Acupuncture, China Medical University, Taichung, Taiwan.

**Keywords:** acupressure, hyperbaric oxygen therapy, middle ear barotrauma

## Abstract

**Background:**

In hyperbaric oxygen therapy (HBOT), a patient is exposed to pure oxygen in a chamber. While HBOT is a long-standing and well-established treatment for a wide variety of medical conditions, one of the main complications is middle ear barotrauma (MEB), which can lead to complaints of ear discomfort, stuffiness or fullness in the ear, and difficulties in equalizing ear pressure. The aim of this study is to evaluate the efficacy of self-acupressure in preventing and reducing the degree of MEB associated with HBOT.

**Methods:**

This is a prospective nonrandomized controlled study. A sample of 152 participants will be assigned to 2 groups in a 1:1 ratio. The participants in the control group will receive conventional Valsalva and Toynbee maneuvers, while those in the experimental group will be given additional self-acupressure therapy. The acupoints used will be TE17 (Yifeng), TE21 (Ermen), SI19 (Tinggong), and GB2 (Tinghui). The Modified Teed Classification, symptoms of MEB, and overall ear discomfort levels will be assessed. Data will be analyzed using the Chi-Squared test or *t* test.

**Objectives:**

This study aims to evaluate the efficacy of self-acupressure for preventing and reducing the degree of MEB associated with HBOT.

**Trial registration:**

ClinicalTrials.gov Identifier: NCT04311437. Registered on 17 March, 2020.

## 1 Introduction

Hyperbaric oxygen therapy (HBOT) is a treatment in which a patient is exposed to pure oxygen for a period of time and under a certain pressure in a single-patient (monoplace) or multiple-patient (multiplace) chamber.^[1,2]^ For clinical purposes, the Undersea and Hyperbaric Medical Society (UHMS) indicates that pressurization should be 1.4 atmospheres absolute (ATA) or higher to be effective. The pressure range between 2 and 3 ATA is normally used for therapeutic purposes.^[3]^ In a monoplace chamber, only 1 patient breathes in directly pressurized 100% oxygen, while in a multiplace chamber, several patients can breathe pressurized 100% oxygen indirectly via head hood, mask, or endotracheal tube.^[4]^

HBOT is a long-standing and well-established treatment for a wide variety of medical conditions, such as musculoskeletal injuries, infections, poor wound healing, hypoxia in neurons, carbon monoxide poisoning, decompression illness, and gas embolisms.^[2,4–7]^ Although HBOT is used in numerous pathological processes and is generally safe and well tolerated, certain effects and complications may occur during the treatment,^[8]^ some examples being middle ear barotrauma (MEB), claustrophobia, oxygen toxicity, myopia, pulmonary dyspnea, and oxygen-induced seizures.^[9]^ Among all the side effects of HBOT, MEB is a common one,^[10]^ with an incidence of approximately 2%.^[9]^ However, MEB is difficult to diagnose directly. Instead, it is mostly based on patients’ subjective reports of symptoms such as ear discomfort, stuffiness or fullness in the ear, and difficulties in equalizing ear pressure.^[11]^ Once a patient exhibits clinical signs and symptoms of MEB, s/he must be evaluated via otoscopic examination to diagnose and classify the extent of the injury. Only with proper grading of MEB can medical staff decide whether treatment is required or not. Treatments can take the form of enhanced equalization education, medical intervention, or surgical intervention such as myringotomy and ventilation tube placement. Currently, MEB is evaluated and graded with 3 methods: the Teed, Modified Teed, and O’Neill grading systems.^[12]^ It is hard to predict which patients may have difficulty equalizing middle ear pressure during actual hyperbaric exposure. In clinical practice, several methods are proposed to prevent MEB associated with HBOT. These include the Valsalva and Toynbee maneuvers to open the Eustachian tubes and facilitate pressure equalization,^[13]^ nasal decongestants to reduce hyperemia and edema of the middle ear mucosal lining,^[14]^ and prophylactic myringotomy or tympanostomy tube placement.^[15]^ To the best of the authors’ knowledge, no significant studies published to date have evaluated MEB prevention methods during clinical hyperbaric exposures.

Acupuncture is a technique in traditional Chinese medicine (TCM) that was originally thought to work on the principle of redistribution of Qi, the life energy.^[16]^ In TCM theory, disease is believed to originate from an imbalance or poor flow of Qi.^[16]^ A treatment that is related to acupuncture, acupressure, is a form of touch therapy that utilizes the principles of acupuncture and Chinese medicine.^[16,17]^ In acupressure, the same anatomic locations are used as in acupuncture, but they are stimulated with finger pressure instead of needle insertion. As opposed to acupuncture, acupressure is noninvasive, simple, and typically painless. Additionally, adverse reactions caused by the insertion of fine needles can be avoided. Self-acupressure is acupressure performed by trained participants without treatment by practitioners or healthcare providers. Self-acupressure can be administered in any situation, regardless of time and place, and self-treatment can be conducted without expensive costs. Currently, several systemic reviews have reported positive effects of self-acupressure for symptom management, including symptoms of constipation, allergic diseases, nausea, vomiting, pain, stress, and sleep disturbance.^[17–20]^ So far, no research on acupressure for HBOT-related MEB has been conducted. Therefore, the aim of this study is to evaluate the efficacy of self-acupressure for preventing and reducing the degree of MEB associated with HBO.

## 2 Methods/design

### 2.1 Ethics approval

This protocol has been reviewed and approved by the Institutional Review Board of the Chang Gung Medical Foundation (IRB no. 201901443B0; version 3 on October 21, 2019). The protocol identification number at https://clinicaltrials.gov is NCT04311437. This study will be conducted in accordance with the principles of the Declaration of Helsinki. Before participation, all patients will receive both verbal and written forms of detailed information about the trial from physicians of the HBOT center at Kaohsiung Chang Gung Memorial Hospital (KCGMH). All participants will voluntarily sign informed consent forms that have been approved by the ethics committee prior to enrollment. Personal information about potential and enrolled participants will be collected, shared, and maintained in an independent closet to ensure confidentiality before, during, and after the trial. We will present the results and submit them for publication in peer-reviewed journals. JMC and CTL will have access to the final trial dataset, as well as disclosure of contractual agreements that limit such access for investigators.

### 2.2 Study design

**Figure 1:**
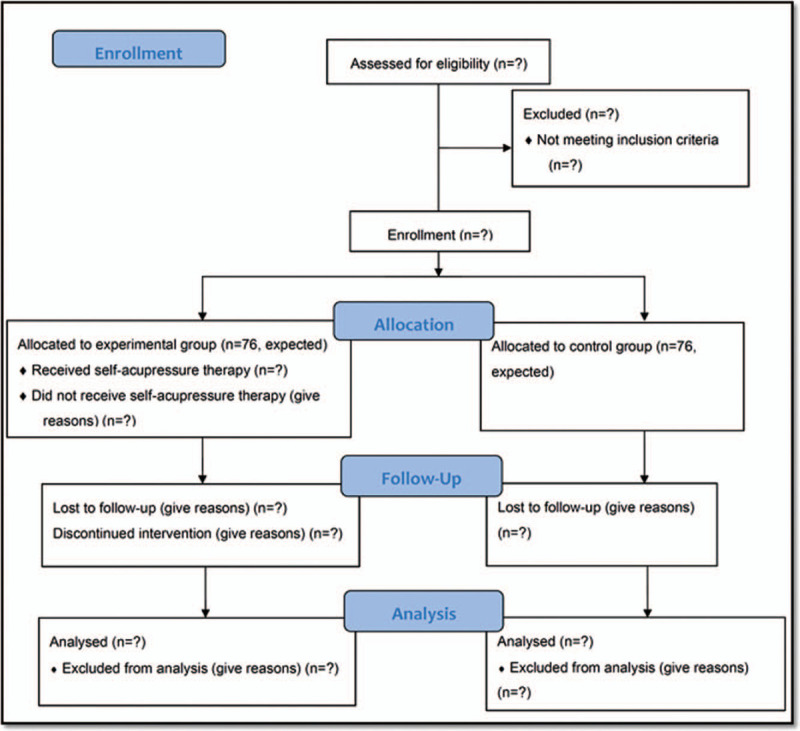
The flowchart of the trial.

This prospective nonrandomized controlled trial began at the HBOT center at KCGMH in southern Taiwan in March 2020 and will continue through February 2022. The recruited participants will be assigned to the experimental group (conventional Valsalva and Toynbee maneuvers plus self-acupressure therapy, n = 76, expected) or the control group (conventional Valsalva and Toynbee maneuvers, n = 76, expected). All participants will receive education on the Valsalva and Toynbee maneuvers before the first HBOT. Participants in the experimental group will receive additional self-acupressure education from the same trained practitioner before the first HBOT. Because participants could learn the technique of self-acupressure from each other while receiving HBOT in the same chamber, the participants in the experimental and control groups will be recruited separately in different time periods. The study design has been written in accordance with the Consolidated Standards of Reporting Trials (CONSORT) 2010 guidelines.^[21]^ The overall schematic chart is provided in Figure 1.

### 2.3 Participants

The source of participant recruitment is the HBOT center at KCGMH. Physicians at the HBOT center provide information about this study to participants and then assess the participants’ eligibility to participate in it. Participants who agree to participate in the trial will provide written consent after receiving an oral explanation of the objectives and procedures of the study from their physicians.

#### 2.3.1 Inclusion criteria

Subjects are eligible to participate if they meet all the following criteria:

1. age of 20 years or older;
2. clear consciousness and ability to communicate;
3. prescription to receive first HBOT;
4. consent to participate in the study.

#### 2.3.2 Exclusion criteria

Subjects will be excluded if they meet any of the following criteria:

1. pregnancy;
2. acute disorders of the ears or upper respiratory tract;
3. evidence of neurologic dysfunction precluding them from making an informed decision;
4. tracheostomy or endotracheal intubation;
5. myringotomy, tympanoplasty, mastoidectomy, or tympanostomy tube placement.

#### 2.3.3 Dropout criteria

Participants will be dropped from the trial under the following conditions:

1. unstable vital signs and need for first aid during study period;
2. deterioration in clinical condition leading to an assessment by medical staff of unsuitability to continue the study;
3. participant's decision to withdraw from the trial at any time.

### 2.4 Sample size calculation

According to a previous study on the effect of dried salted plum for HBOT induced otalgia,^[22]^ moderate to severe otalgia was present in 0.0% (0 of 53) of those consuming a dried salted plum vs 10.8% (4 of 37) of the control group (*P* = .026). Anticipating a power of 80% (1 – β = 0.8), statistical significance (α = 0.05) of 95%, and a dropout rate of 10%, a total of 76 participants for each group will be required in this study (https://clincalc.com/stats/samplesize.aspx).

### 2.5 Interventions

**Figure 2:**
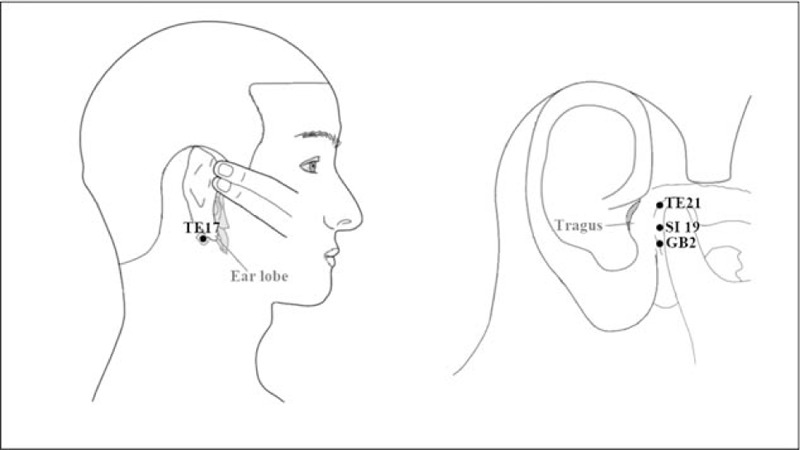
Acupoints used in the self-acupressure therapy: TE17, TE21, SI19, and GB2.

All participants will receive education from trained practitioners on the Valsalva and Toynbee maneuvers before the first HBOT. The participants in the experimental group will undergo additional education and training in self-acupressure therapy. The acupoints used will be TE17 (Yifeng), TE21 (Ermen), SI19 (Tinggong), and GB2 (Tinghui) (Fig. 2). All acupoints have been selected and localized according to the WHO Standardized Acupuncture Point Location guidelines.^[23]^ The education on self-acupressure therapy will be provided by the same trained practitioner, who has received sufficient training from a licensed TCM doctor in Taiwan. The self-acupressure therapy protocol is as follows: In a sitting position, the patient applies firm pressure (3–5 kg of pressure) with the fingertips in a circular motion at a speed of 2 circles per second for a duration of one minute per acupoint, with 1 to 2 seconds of rest after each 10 circles. The complete process lasts for about 5 minutes. A weighing scale is used for calibrating the correct amount of finger pressure necessary for the acupressure treatment. The participants in the control group will receive routine Valsalva and Toynbee maneuvers before the first HBOT, without additional education on self-acupressure.

### 2.6 Outcome measurements

The primary outcome measurement is the presentation of the tympanic membrane recorded according to the Modified Teed Classification, with grades from 0 to 5, where Grade 0 indicates symptoms with no ontological signs of trauma; Grade 1 indicates injection of the tympanic membrane; Grade 2 indicates Grade 1 plus injection plus mild hemorrhage within the tympanic membrane; Grade 3 indicates gross hemorrhage within the tympanic membrane; Grade 4 indicates free blood in the middle ear, evidenced by blueness and bulging; and Grade 5 indicates perforation of the tympanic membrane.

The secondary outcomes are symptoms of MEB and the overall ear discomfort levels. Recorded symptoms of MEB include feeling pressure in the ears, ear pain, headache, dizziness, vertigo, tinnitus, and hearing loss. The symptoms of MEB and the overall ear discomfort levels will be estimated using a 10-cm visual analogue scale with anchor points of 0 (no discomfort) and 10 (maximum discomfort) at the same time as the primary outcome measure. A higher score will indicate a higher level of discomfort during HBOT. The outcomes will be measured at baseline before the first HBOT, after the first 5 consecutive treatments of HBOT, and after each 10 treatments of HBOT until the twentieth HBOT.

### 2.7 Statistical analysis

Quantitative variables, presented as mean ± SD, will be analyzed by the Student *t* test. Qualitative variables, expressed as a number (percentage), will be analyzed using the Chi-Squared test. All analyses will be performed in SPSS software. Differences will be considered statistically significant at *P* < .05.

### 2.8 Data monitoring

No data monitoring committee will be needed because self-acupressure is general practice and a noninvasive intervention. Nevertheless, adverse events such as subcutaneous hematomas or localized rash near the location of self-acupressure therapy will be monitored during the trial period.

## 3 Discussion

MEB, one of the main common side effects of HBOT, is a major reason why patients discontinue HBOT. Although the Valsalva and Toynbee maneuvers are usually used to prevent symptoms of MEB associated with HBOT, many patients still experience ear discomfort during and after HBOT. The present study aims to evaluate whether additional self-acupressure in combination with the Valsalva and Toynbee maneuvers is more effective in preventing and reducing the discomfort caused by MEB than are the Valsalva and Toynbee maneuvers alone. Self-acupressure is a simple, noninvasive treatment that can be performed at any time, as opposed to acupuncture, which is invasive and can only be performed by trained physicians. If additional self-acupressure is proven more effective, patients may be more willing and feasible to complete the course of HBOT.

Acupressure, which originated in ancient China, is based on the theory of TCM and is closely associated with acupoint activation across the meridians, just as acupuncture is. Acupressure uses pressure to stimulate specific acupoints which can correct imbalances in the *Qi* through channels and subsequently treat diseases.^[16]^ The biochemical mechanism of acupressure involves the stimulation of acupoints that leads to complex neuro-hormonal responses, such as antiinflammation via the hypothalamic-pituitary-adrenocortical axis.^[16]^ In addition, acupressure mediates the nitric oxide signal, which is known to improve local microcirculation via cyclic guanosine monophosphate.^[16]^ Stimulation of acupoints near the ear may alleviate symptoms of MEB. In the present study, correct position and proper strength during self-acupressure will be important. One well-trained practitioner will educate the participants in the experimental group on additional self-acupressure therapy and confirm that the self-acupressure is correctly performed. The participants in the experimental group and those in the control group will be recruited in separate periods of time so as to avoid contact between the 2 groups of participants and thereby minimize the likelihood that the patients might learn about self-acupressure from one another while receiving HBOT in the same chamber.

In conclusion, the results of this study are expected to provide evidence on the efficacy of self-acupressure as a no cost, noninvasive self-management method for MEB associated with HBOT in addition to the Valsalva and Toynbee maneuvers.

## Acknowledgments

The authors thank the Biostatistics Center, Kaohsiung Chang Gung Memorial Hospital, for statistics work.

## Author contributions

**Conceptualization:** Kuo-Wei Bi, Chun-Ting Liu.

**Data curation:** Re-Wen Wu.

**Formal analysis:** Zheng-Nan Lu.

**Writing – original draft:** Jen-Ming Chen.

**Writing – review & editing:** Chun-Ting Liu.
